# Radiation-Induced Sarcoma Originating in Recurrent Juvenile Nasopharyngeal Angiofibroma

**DOI:** 10.1155/2018/5695803

**Published:** 2018-07-15

**Authors:** Smriti Panda, Madhu Rajeshwari, Chirom Amit Singh, Suresh C. Sharma, Pirabu Sakthivel

**Affiliations:** ^1^Department of Otolaryngology & Head & Neck Surgery, All India Institute of Medical Sciences, New Delhi, India; ^2^Department of Pathology, All India Institute of Medical Sciences, New Delhi, India

## Abstract

Juvenile nasopharyngeal angiofibroma is a benign disease affecting young males with a propensity to invade intracranially and into the orbit along preformed pathways. Complete surgical excision is the mainstay of management. Patients with multiple recurrences along with tumour extension into skull base and orbit can be considered for external beam radiation as either adjuvant or definitive treatment. Possibility of radiation-induced malignancy has been speculated by many authors, proof of which exists in only two studies so far. This report focuses on radiation-induced leiomyosarcoma in a patient with recurrent juvenile nasopharyngeal angiofibroma.

## 1. Introduction

Juvenile nasopharyngeal angiofibroma is a benign tumour originating in the nasopharynx, presenting in adolescent males with nasal mass, epistaxis, nasal obstruction, cheek swelling, and proptosis. Though benign, it has a capacity for local invasion along preformed pathways. Thus, JNA can encroach into critical structures like optic nerve, internal carotid artery and present intracranially. Surgery is the main modality of treatment, preceded by embolisation of arterial feeders in case of extensive tumours involving infratemporal fossa or intracranial involvement. For tumours infiltrating into infratemporal fossa, sphenoid sinus, base of pterygoid, cavernous sinus, foramen lacerum, anterior and middle cranial fossa [1], subtotal tumour excision predisposes to recurrence. Patients with massive intracranial involvement, incomplete resection, and recurrent tumours can be considered for either definitive radiotherapy or radiotherapy in the adjuvant setting [2]. However, use of radiation in the case of a benign tumour in such young patients should be weighed against the possible complications like osteoradionecrosis, panhypopituitarism, temporal lobe necrosis, cataract, radiation keratopathy, and rarely radiation-induced malignancy [3]. This is a report of a case of recurrent JNA postmultiple surgeries developing radiation-induced leiomyosarcoma 3 years after receiving radiation therapy.

## 2. Case Report

Our patient is a 37-year-old male who was previously diagnosed as a case of juvenile nasopharyngeal angiofibroma. He was diagnosed with the condition at 30 years of age during which he underwent his first surgery by the lateral rhinotomy approach. Tumour was seen to involve the nasopharynx and sphenoid sinus, eroding the basisphenoid. Optic nerve and carotid artery were not involved. He underwent endoscopic excision 2 years later for tumour involving nasopharynx and sphenoid sinus. The patient remained symptom-free for 2 years, following which lateral rhinotomy excision was performed for a recurrent tumour involving nasopharynx, sphenoid sinus, eroding basisphenoid, and vidian canal. Postoperative histopathology confirmed the presence of angiofibroma and ruled out the presence of any sarcomatous element. In view of the frequently recurring nature of the tumour, the patient was given 45 Gy, 25 fractions of conformal radiotherapy. During the routine follow-up nasal endoscopy after 3 years, a fleshy vascular mass was seen in the nasopharynx. The patient underwent subtotal excision at another centre by lateral rhinotomy.

**Figure 1:**
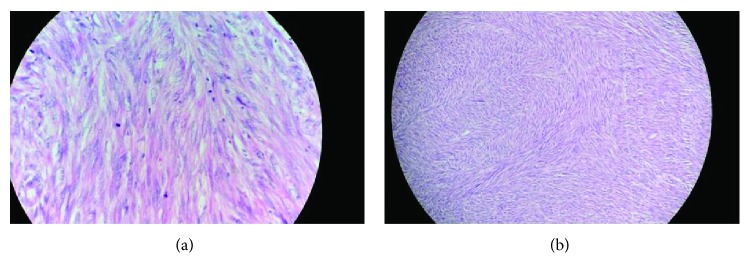
Histopathology sections showing spindle-shaped tumour cells arranged in long intersecting fascicles, with moderate nuclear pleomorphism and increased mitosis.

The specimen block was reviewed. Sections examined showed spindle-shaped tumour cells arranged in long intersecting fascicles, with moderate nuclear pleomorphism and increased mitosis (Figure 1). Tumour cells were immunopositive for smooth muscle actin, while negative for CD34, cytokeratin, CD 56, S100, and HMB 45. MIB-1 labelling index was 25%. Overall features were suggestive of leiomyosarcoma.

**Figure 2:**
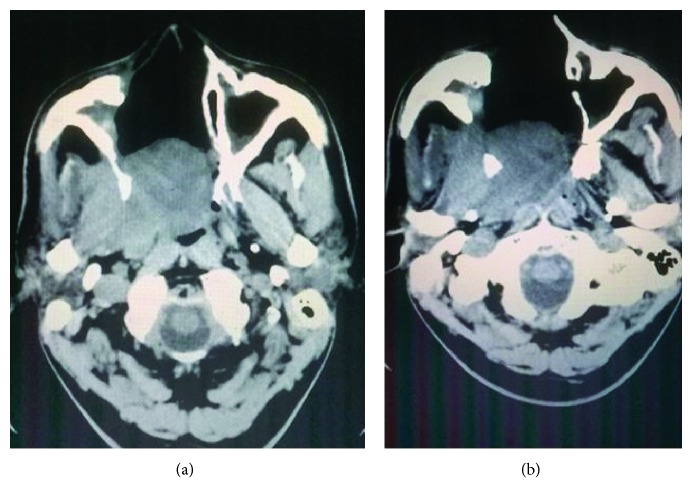
Enhancing soft tissue mass arising from nasopharynx, destroying pterygoid plates and extending to infratemporal fossa.

Contrast-enhanced computed tomogram showed heterogeneously enhancing soft tissue mass lesion in the right posterolateral wall of nasopharynx measuring 6 × 3.7 × 4 cm with erosion of the adjacent bone with involvement of the pterygoid muscles (Figure 2).

In view of the extensive involvement of skull base, surgery was not considered to be suitable for providing a negative margin. Patient has thus been planned for neoadjuvant chemotherapy (gemcitabine based) followed by chemoradiation.

## 3. Discussion

Surgery is undoubtedly the definitive treatment option for juvenile nasopharyngeal angiofibroma. Dilemma arises when there are multiple recurrences, extensive intracranial, intraorbital, or infratemporal fossa involvement. There are multiple case series and case reports citing the use of radiation therapy for this benign condition. Cure rates of 80% have been reported for both these modalities of treatment [2]. The recent largest series is that of 32 patients of stage III JNA from India, reviewed over a period of 25 years [4]. Median dose of radiotherapy was 30 Gy, with 14 patients treated with a radical intent. The same authors reported a case of squamous cell carcinoma of the nasal ala developing 15 years after receiving radiotherapy. Notable complications following radiation therapy for JNA reported in the literature include panhypopituitarism, posterior capsular opacification, temporal lobe necrosis, growth retardation, and radiation keratopathy [5]. Numerous other studies have evaluated the role of radiation therapy for JNA [2, 6–9], of which secondary malignancies were reported by Cummings et al. [2] (thyroid carcinoma and basal cell carcinoma of skin, 14 and 13 years after irradiation, resp.) and Reddy et al. [8] (basal cell carcinoma). Conley et al. also reported a case of orbital squamous cell carcinoma [10]. Only two studies have reported sarcomatous change in JNA postirradiation [11, 12], though possibility of the same have been put forth by many studies [4, 13]. As in our patient, radiotherapy has been used in the adjuvant setting as well. Álvarez et al. [1] used stereotactic radiosurgery on residual JNA, showing tumour decrement in 3 out of 7 patients after a follow-up of 3 years.

Radiation-induced malignancy (RIM) is thus the rarest and the most feared complication following irradiation for a benign condition like JNA. Our patient fulfilled the criteria for RIM as stated by Cahan et al. [14] and Murray et al. [15]:

1. The tumour arose in a previously irradiated field.
2. The new tumour is histologically different from the original condition.
3. There was no evidence of the new tumour at the time of radiation therapy.
4. A latency period existed between irradiation and the development of the new tumour.

RIM in head and neck has an incidence of 0.9% and develops after a mean latency of 22 years with a mean radiation dose of 6035 cGy, most commonly located in paranasal sinuses [16]. The same study showed that sarcomas outnumbered other RIM histological subtypes. Other studies, however, showed pharynx to be the most common location for RIM and squamous cell carcinoma and thyroid cancers being the most common RIMs [17, 18]. There are studies that claim radiation-induced sarcomas to be more sinister in their course and having shorter latency than other RIM [19, 20]. Studies evaluating risk of RIM in intermediate dose radiation given to benign diseases indicate a very small risk but have advised caution in pediatric population in view of long latency of these tumours [21].

The mechanism behind the occurrence of RIM is not well established. One hypothesis as proposed by Brenner and Shuryak et al. [22, 23] include generation of radiation-induced premalignant cells that initially survive low-dose radiation exposure and subsequently repopulate “sterilized area” following radiotherapy. Due to the small number of affected individuals and long latency period associated with RIM, there is currently no level 1 evidence analyzing risk factors predicting the development of RIM. However, McKeown et al. [21] have reviewed the existing literature and have found a trend towards increased risk of RIM with increasing dose, estimated to be 10%/Sv for high-dose rate exposure. The effect was more pronounced in children. That is, the risk of RIM was 15%/Sv in the case of children < 10 years compared with 1%/Sv in the case of adults > 60 years of age.

Surgery is generally the preferred modality for RIM; however, as in our case due to the involvement of critical structures like skull base, nonsurgical modality including radiation have also been employed for management by other authors [24]. But in general, surgically treated patients have better survival outcomes [17, 24].

Though current radiation techniques have been significantly refined in terms of limiting exposure to uninvolved tissues and putative organs at risk, it is essential to bear in mind this small but potentially life-threatening risk while irradiating benign diseases in the case of children.

## 4. Conclusion

Sarcomatous change in JNA following irradiation is the rarest and the most dangerous complication ever reported. This case report aims to lay emphasis on this aspect of managing JNA which recur frequently. Multiple recurrences and skull base involvement precludes surgery as an option in our patient. Though both the modalities of treatment have equal recurrence rates, morbidity and mortality, use of conformal radiotherapy should be carefully weighed against the possible occurrence of such complications. The long-term effects of IMRT and stereotactic radiosurgery also need to be evaluated in detail before these modalities can be used in this setting without being fraught with such complications.
